# A negative binomial latent factor model for paired microbiome sequencing data

**DOI:** 10.1186/s12859-025-06362-3

**Published:** 2026-01-22

**Authors:** Hyotae Kim, Nazema Y. Siddiqui, Lisa Karstens, Li Ma

**Affiliations:** 1 Department of Biostatistics & Bioinformatics, Duke University, 2424 Erwin Road, Durham, NC 27705 USA; 2 Department of Obstetrics and Gynecology, Duke University, 203 Baker House, Durham, NC 27710 USA; 3 Department of Medical Informatics and Clinical Epidemiology and Department of Obstetrics and Gynecology, Oregon Health & Science University, 3181 S.W. Sam Jackson Park Road, Portland, OR 97239 USA; 4 Department of Statistics and Data Science Institute, University of Chicago, 5747 S Ellis Avenue, Chicago, IL 60637 USA

**Keywords:** Bayesian modeling, Latent factor models, Paired microbiome sequencing data, Pólya-Gamma augmentation

## Abstract

**Background:**

Microbiome sequencing data are often collected from several body sites and exhibit dependencies. Our objective is to develop a model that enables joint analysis of data from different sites by capturing the underlying cross-site dependencies. The proposed model incorporates (i) latent factors shared across sites to explain common subject effects and to serve as the source of correlation between the sites and (ii) mixtures of latent factors to allow heterogeneity among the subjects in cross-site associations.

**Results:**

Our simulation studies demonstrate that stronger associations between two sites lead to greater efficiency loss in regression analysis when such dependence is ignored in modeling. In a case study involving samples collected from a study on the female urogenital microbiome with aging, our model leads to the detection of covariate associations of the vaginal and urine microbiomes that are otherwise not statistically significant under a similar regression model applied to the two sites separately.

**Conclusions:**

We propose a latent factor model for microbiome sequencing data collected from multiple sites. It captures the presumptive underlying cross-site associations without compromising estimation accuracy or inference efficiency in the absence of such associations. In addition, our proposed model improves predictive performance by enabling the prediction of microbial abundance at one site based on observations from another. We also provide an extended framework that allows for clustering of subjects (samples) and cluster-specific levels of paired association. Under this extended framework, clusters can be classified according to their association strengths.

## Background

The advent of next-generation sequencing (NGS) technology enables the identification of a wide range of microbes from environmental samples without the need for cultivation, thus facilitating the exploration of microbial communities. The two most widely used methods for sequencing microbial communities are universal marker gene amplicon sequencing, such as the 16 S rRNA gene, and whole metagenome shotgun sequencing (WMS) of all microbial genomes. These methods produce sequencing reads, which are subsequently mapped to taxa at various taxonomic levels using bioinformatic preprocessing pipelines, such as DADA2 [1] and MetaPhlAn [2]. As a result of this taxonomic profiling, a large, highly sparse count table of taxa per sample is produced, with typically a finer taxonomic resolution in WMS than in 16 S amplicon sequencing.

To gain a comprehensive understanding of the human microbiome, researchers often collect data from multiple body sites for each individual and compare the composition and functions of the microbial communities in different parts of the body (e.g., [3, 4]). The UMICRO data set, which we will use as a case study, includes vaginal-urine paired samples obtained by vaginal swabbing of the distal vagina and urine collection by transurethral catheterization, with the aim of identifying the microbial composition of the two communities and analyzing their variations with respect to the menopausal status of the participants. As the sample pairs were collected from two different body sites of the same subject, common effects associated with the subject are likely to be present in both vaginal and urine samples. This data set motivates the development of a model to capture the potential associations between the vaginal and urine niches.

For the analysis of microbial sequencing data in the form of a count table with rows for samples and columns for taxa, modeling methods originally developed for RNA sequencing (RNA-seq) or single-cell RNA sequencing (scRNA-seq) data can be adopted. Similar to microbial data, RNA sequencing data provide sparse count tables that report the number of sequence fragments assigned to each gene per sample or cell, for which the following models have been devised: negative binomial models [5, 6], zero-inflated negative binomial models [7], Poisson zero-inflated log-normal models [8], and truncated Gaussian hurdle model [9]. In addition, [10–13] introduced beta-binomial, multinomial, and zero-inflated Gaussian models, which were specifically developed for microbiome data. Although zero-inflated models were created to accommodate the pronounced sparsity of microbiome data, their validity in count-based models for sequencing reads remains controversial. As discussed in [14, 15], in many common settings involving sparse sequencing count data, the abundance of zeros can often be adequately accommodated by simply incorporating overdispersion into count-based sampling models, such as in negative binomial models, without needing an additional zero-inflation component. We share this viewpoint, and because we are considering overdispersed count-based models in this paper, we do not by default incorporate an additional zero-inflation component, but in cases where such a component is indeed justified, incorporating it into the model is straightforward.

We propose a modeling framework to jointly analyze microbiomes from two (or more) body sites, adopting a negative binomial regression model for each site while incorporating a shared latent factor to parsimoniously capture potential correlation in paired samples from different body sites in some taxa. Specifically, two negative binomial distributions, one for each site, share a set of latent factors, which are interpreted as unobserved common effects that contribute to their correlation. This paper focuses on a paired two-site data set, for which the latent factor model corresponds to a mixed-effects model. The framework can be generalized to more than two sites by introducing multiple latent factor vectors with associated factor loadings.

Mixed-effects models are commonly used for repeated-measurement data. Zhang et al. [16] developed a zero-inflated Gaussian mixed model for longitudinal microbiome data (see [17–19] for other linear (Gaussian) mixed-effects applications in microbiome analysis). However, Gaussian-based models require transforming count data to the real line, which can introduce bias and make the results highly sensitive to the choice of transformation. We demonstrate this later in the model comparison, using a log transformation with several choices of pseudocounts. Chen and Li [20] proposed a beta–distribution-based mixed model with two regression components: one for the probability of observing zero abundance (i.e., presence/absence modeling) and the other for relative abundance among nonzero observations. In this formulation, a regression coefficient no longer represents the overall effect of a covariate on abundance but instead corresponds to separate effects for “presence/absence” and “abundance conditional on presence”. In addition, the number of parameters doubles, increasing the complexity of inference. More importantly, both mixed-effects approaches model relative abundances, which induce compositionality and render the independence assumption across taxa untenable. In contrast, our approach models count data directly, requiring no transformation and allowing independence across taxa to be reasonably assumed, which enables independent modeling and parallel implementation for inference.

We extend our model to allow the latent factors to form subgroups, each of which can exhibit distinct cross-site correlation patterns. We present versions of the model that work both when the subgrouping is observed and when it is not. In the latter case the subgrouping is inferred from the data. We carry out a case study on the UMICRO data, where the subjects are categorized into three subgroups according to menopausal status.

The rest of the paper is organized as follows. Section 2 introduces our negative binomial latent factor model with Sect. 2.1 for the base modeling framework and Sect. 2.2 for some model extensions. In Sect. 2.3, we present a hierarchical representation of our model with prior specifications, followed by a posterior prediction approach. The proposed model is illustrated through synthetic data in Sect. 3.1 and the UMICRO study in Sect. 3.2. Finally, Sect. 4 concludes.

## Methods

### Joint negative binomial model

For a given taxon (e.g., genus), we use $y_{si}$ to denote the observed count in sample *i* at body site *s*, with $i=1,\ldots ,n$ and $s=1,2$. Although the data are also indexed by taxa, we suppress the taxon index throughout for simplicity, as the model is taxon-specific and applied separately to each taxon. Let $\text {NB}(\mu ,\alpha )$ be a negative binomial distribution with mean $\mu$ and variance $(1+\mu \alpha )\mu$. We consider the following joint negative binomial model (JNBM) that relates the counts from two sampling sites through a taxon-specific latent factor, $\gamma _{i}$, in the form of a multiplicative factor on the mean count for the taxon,

1 $$\begin{aligned} \begin{aligned} y_{1i}&\overset{\textit{ind.}}{\sim }\text {NB}(\mu _{1i},\alpha _1), \quad \mu _{1i} = \exp (\gamma _{i}) N_{1i} \exp (X_i'\beta _1)\\ y_{2i}&\overset{\textit{ind.}}{\sim }\text {NB}(\mu _{2i},\alpha _2), \quad \mu _{2i} = \exp (\gamma _{i}) N_{2i} \exp (X_i'\beta _2)\\ \gamma _{i}&\overset{\textit{i.i.d.}}{\sim }\text {N}(-\phi ^2/2,\phi ^2).\\ \end{aligned} \end{aligned}$$

Here, the notation $\overset{\textit{ind.}}{\sim }$ represents “independently distributed” and $\overset{\textit{i.i.d.}}{\sim }$ represents “independent and identically distributed”. $X_i$ denotes the vector of covariates for sample *i*, and $\beta _{s}$ is the regression coefficient vector for body site *s*. $\alpha _{s}$ is the overdispersion parameter of the negative binomial distribution. $N_{si}$ indicates the total number of read counts, that is, the sum of $y_{si}$ over all taxa of interest. The latent factor $\gamma _{i}$ $\big ( \text {or its exponential}\exp (\gamma _{i})\big )$ is an unobserved variable representing a site-invariant, sample-specific effect, such as unobserved clinical or demographic characteristics of participants. The multiplicative effect of $\exp (\gamma _{i})$ on the mean, $\mu _{si}$, has $\text {E}(\exp (\gamma _{i}))=1$ and $\text {Var}(\exp (\gamma _{i})) = \exp (\phi ^2)-1$. This distributional assumption with the mean of 1 enables the random effects of $\exp (\gamma _{i})$ to have no preference for a positive impact $(>1)$ or negative impact $(<1)$. The parameter $\phi ^2$ measures the strength of the association between the two body sites; when $\phi ^2$ becomes zero, the model is reduced to two independent negative binomial distributions for each body site, that is, $\text {NB}(N_{si}\exp (X_i'\beta _{s}),\alpha _{s})$ for $s=1,2$. We will refer to it as the separate negative binomial model (SNBM). Section 3.2 will compare our joint two-site model with SNBM in a case study on the UMICRO data.

To perform JNBM-based Bayesian inference, we need priors for $(\alpha _{s},\beta _{s},\phi ^2)$; a description of the complete Bayesian hierarchical model can be found in Section 2.3.1. A fully conjugate sampling recipe for posterior inference based on the Pólya-Gamma data augmentation technique is detailed in Supplementary Material B.

For brevity in the model description, we assume balanced paired data, i.e., both sites have the same number of samples. However, the model can also be used for unbalanced data where some samples are available only for one site.

### Model extension

Although JNBM assumes a constant level of cross-site association throughout all paired samples, in real-world examples, this assumption is often unrealistic. However, allowing each sample to have its own level of association may lead to an overly flexible model. We thus compromise and assume that there are subgroups (either observed or unobserved) among the samples for which the extent of cross-site association is comparable. Consequently, we extend the base joint model by allowing different sample groups to exhibit different levels of paired association, which can easily be achieved by replacing the normal distribution for the latent factors with a mixture distribution. The following is a two-component normal mixture-based model, which randomly assigns paired samples to two groups:

2 $$\begin{aligned} \gamma _{i} \overset{\textit{i.i.d.}}{\sim }\nu \text {N}(-\phi ^2_1/2,\phi ^2_1) + (1-\nu )\text {N}(-\phi ^2_2/2,\phi ^2_2), \qquad \phi ^2_1 < \phi ^2_2. \end{aligned}$$

It should be noted that we enforce an inequality constraint on the mixing parameter, $\phi ^2_{1}$ and $\phi ^2_{2}$, to ensure model identifiability. The general form of the mixture prior is given by $\sum _{l=1}^L \nu _{l} \text {N}(-\phi ^2_{l}/2,\phi ^2_{l})$ with $0<\phi ^2_1<\ldots <\phi ^2_{L}$ and $\sum _{l=1}^L \nu _{l}=1$ for $L>1$. In the paper, we will call this joint negative binomial model with the two-component mixture distribution JNBM_Mix. Regarding the choice of *L*, we recommend starting with an initial value (e.g., $L = 3$ or 4) and conducting a sensitivity analysis by gradually increasing *L* until the number of estimated clusters containing at least one sample is less than *L* for all taxa. Supplementary Material A.4 presents random clustering results for the Lactobacillus samples as an example. It also provides a table from the sensitivity analysis of the number of estimated clusters under $L=4$ and $L=8$, supporting our choice of $L=8$.

Alternatively, the clustering of paired samples can be accomplished by using a given variable $g(i)=\{1,\ldots ,L\}$ rather than random assignment with $\{\nu _{l}\}$, where differences in association levels across *L* groups may serve as an indicator of the proximity among them. The mixture distribution for $\gamma _{i}$ is adjusted as follows:

3 $$\begin{aligned} \gamma _{i} \overset{\textit{ind.}}{\sim }\sum _{l=1}^L \delta _l\Big (g(i)\Big ) \text {N}(-\phi ^2_{l}/2,\phi ^2_{l}) = \text {N}(-\phi ^2_{g(i)}/2,\phi ^2_{g(i)}), \end{aligned}$$

where $$\delta _l(x)$$ is a delta function with $$\delta _l(x) = 1$$ for $$x=l$$ and 0 otherwise. For the UMICRO case study, we use Study Group variable for clustering, such that $$g(i) = $$ {Postmenopausal with no estrogen, Postmenopausal on estrogen, Premenopausal}, which model is named JNBM_SG.

### Posterior inference

#### Hierarchical model representation

Below is a full description of the joint negative binomial model (JNBM) presented in (1) of Section 2.1. Let $\boldsymbol{y} = \{y_{si}: s=1,2 \text { and } i=1,\ldots ,n\}$, $\boldsymbol{\gamma } = \{\gamma _{i}: i=1,\ldots ,n\}$, and $\beta _{s}=\{\beta _{sp}: p=1,\ldots ,P\}$ for $s=1,2$. The model is defined as

$$\begin{aligned} \begin{aligned} p(\boldsymbol{y}|\{\alpha _{s}\},\{\beta _{s}\},\boldsymbol{\gamma })&= \prod _{s=1}^2\prod _{i=1}^n \Big [\frac{\Gamma (y_{si}+\alpha _{s}^{-1})}{\Gamma (y_{si}+1)\Gamma (\alpha _{s}^{-1})}\Big (\frac{\alpha _{s}^{-1}}{\alpha _{s}^{-1}+\mu _{si}}\Big )^{\alpha _{s}^{-1}}\Big (\frac{\mu _{si}}{\alpha _{s}^{-1}+\mu _{si}}\Big )^{y_{si}}\Big ]\\ \gamma _{i}|\phi ^2&\overset{\textit{i.i.d.}}{\sim }\text {N}(-\phi ^2/2,\phi ^2), \hspace{5.0pt}i=1,\ldots ,n,\\ \beta _{sp}|\tau ^2_s&\overset{\textit{ind.}}{\sim }\text {N}(-\tau ^2_s/2,\tau ^2_s), \hspace{5.0pt}p=1,\ldots ,P, \hspace{5.0pt}s = 1,2,\\ (\alpha _{s},\phi ^2,\tau ^2_s)&\overset{\textit{ind.}}{\sim }\text {Exp}(a_{\alpha })\text {Exp}(a_{\phi ^2})\text {Exp}(a_{\tau ^2}), \hspace{5.0pt}s=1,2,\\ \end{aligned} \end{aligned}$$

where $\mu _{si} = \exp \big (\gamma _{i}+\log (N_{si})+X_i'\beta _{s}\big )$. For simplicity, we place exponential priors on $(\alpha _{s},\phi ^2,\tau ^2_s)$. The rate parameters $(a_{\alpha },a_{\phi ^2},a_{\tau ^2})$ are chosen through sensitivity analysis. Our primary parameters of interest, the regression coefficients, are robust to the choice of these hyperparameters unless they are set to be substantially large, in which case the estimates of $(\alpha _{s},\phi ^2,\tau ^2_s)$ are unduly forced toward zero, which in turn shrinks the regression coefficients toward zero. Accordingly, we began the sensitivity analysis with an arbitrary value and gradually decreased it until the estimates of $\{\beta _{sp}\}$ stabilized. We choose the hyperparameters $(a_{\alpha },a_{\phi ^2},a_{\tau ^2}) = (1,0.1,0.001)$ for the analyses in Section 3, with sensitivity analysis results for the case study provided in Supplementary Material A.6, using *Streptococcus* as an example. Although the posterior distributions of $(\alpha _{s}, \phi ^2, \tau ^2_s)$ are sensitive to the choice of hyperparameters, those of the regression coefficients show little change unless the priors are deliberately set to be concentrated near 0 using larger hyperparameters.

As with $\gamma _{i}$, we specify normal priors for the regression coefficients $\beta _{sp}$ with mean $-\tau ^2_s/2$ and variance $\tau ^2_s$, so that the multiplicative effects of $\exp (\beta _{sp})$ on $\mu _{si}$ have mean 1 and variance $\exp (\tau ^2_s)-1$. Supplementary Material B discusses the augmented likelihood under this negative binomial modeling framework, which enables the regression coefficients and the latent factors to have full conditionals in closed form.

Similarly, we can represent the extended models – JNBM_Mix and JNBM_SG – with variations in distributional assumptions on $\gamma _{i}$ as follows,

$[{\textbf {JNBM}}\_{\textbf {Mix}}]:$ with auxiliary variables $\{\xi _{i}\}$ for a hierarchical representation of the mixture distribution on $\gamma _{i}$,

4 $$\begin{aligned} \begin{aligned} \gamma _{i}|\phi ^2_1,\ldots ,\phi ^2_{L},\xi _{i}&\overset{\textit{ind.}}{\sim }\text {N}(-\phi ^2_{\xi _{i}}/2,\phi ^2_{\xi _{i}}),\quad i=1,\ldots ,n\\ p(\xi _{i}=l|\nu _{l})&= \nu _{l},\quad l=1\,\ldots ,L\\ (\nu _1,\ldots ,\nu _{L})&\sim \text {Dir}(p_{\nu _1},\ldots ,p_{\nu _{L}})\\ \phi ^2_{l}&\overset{\textit{ind.}}{\sim }\text {Exp}(\phi ^2_{l}|a_{\phi ^2_{l}}), \end{aligned} \end{aligned}$$

where $\text {Dir}(p_1,\ldots ,p_L)$ denotes a Dirichlet distribution with $p_l=1/L$.

$$[{\textbf {JNBM}}\_{\textbf {SG}}]:$$

$$\begin{aligned} \begin{aligned} \gamma _{i}|\phi ^2_1,\ldots ,\phi ^2_{L}&\overset{\textit{ind.}}{\sim }\text {N}(-\phi ^2_{g(i)}/2,\phi ^2_{g(i)}),\quad i=1,\ldots ,n\\ \phi ^2_{l}&\overset{\textit{ind.}}{\sim }\text {Exp}(\phi ^2_{l}|a_{\phi ^2_{l}}), \end{aligned} \end{aligned}$$

where *g*(*i*) is an observed variable, e.g., the study group clinical variable of the UMICRO data in Sect. 3.2. We specify the hyperparameter $a_{\phi ^2_{l}}$ of the exponential prior for $\phi ^2_{l}$, with the assumption of $a_{\phi ^2_{1}}=,\ldots ,=a_{\phi ^2_L}$, in the same manner as $a_{\phi ^2}$ of $\phi ^2$ in JNBM.

#### Cross-site prediction

In making predictive inferences about unknown observables $y^*$, we consider the posterior predictive distribution below, from which we draw predictive samples and compute their average. The posterior predictive distribution is

5 $$\begin{aligned} p(y^*|y) = \int p(y^*|\boldsymbol{\theta })p(\boldsymbol{\theta }|y)d\boldsymbol{\theta }, \end{aligned}$$

where *y* denotes the observed data and $\boldsymbol{\theta }$ is a vector of model parameters: $(\alpha ,\beta )$ for SNBM and $(\alpha ,\beta ,\gamma )$ for JNBM. Posterior predictive samples for $y^*$ are drawn from the negative binomial sampling distribution $p(y^*|\boldsymbol{\theta })$ with parameters that are substituted with the posterior samples of $\boldsymbol{\theta }$ taken from $p(\boldsymbol{\theta }|y)$. Then, the mean of the posterior predictive samples becomes the predictive value for $y^*$.

One useful feature of our joint model is the ability to predict counts at one site using observations from the other site. Compared with SNBM, which relies only on $\{y_{s_1i}: i \in A - I\}$, JNBM takes advantage of additional information from $\{y_{s_2i'}: i' \in I\}$ for predictive inference, specifically through posterior sampling of model parameters. By plugging the posterior samples of the model parameters $(\alpha _{s_1},\beta _{s_1},\gamma _{i'})$ into the sampling distribution $\text {NB}\Big (\exp (\gamma _{i'}+\log (N_{s_1i'})+X_{i'}'\beta _{s_1}),\alpha _{s_1}\Big )$ for JNBM (with $\gamma _{i'} = 0$ for SNBM), we obtain posterior predictive samples of $y_{s_1i'}$. Let $\tilde{y}_{s_1i'}^{(b)}$ be a posterior predictive sample from $\text {NB}\Big (\exp (\gamma _{i'}^{(b)}+\log (N_{s_1i'})+X_{i'}'\beta _{s_1}^{(b)}),\alpha _{s_1}^{(b)}\Big )$ with $(\alpha _{s_1}^{(b)},\beta _{s_1}^{(b)},\gamma _{i}^{(b)})$ acquired at *b*-th MCMC iteration for $b = 1,\ldots ,B$. Then, the predictive value $\tilde{y}_{s_1i'}$ for $y_{s_1i'}$ is defined as the posterior mean, that is, $\tilde{y}_{s_1i'} = \sum _{b=1}^B \tilde{y}_{s_1i'}^{(b)}/B$.

In Section 3.2, we evaluate the predictive performance of the models using cross-validation (CV), where zeros are intentionally imposed on the test-set counts to emulate the anomalous zeros that may require prediction in real data. Sequencing depths for the test-set samples are recalculated by subtracting the counts that are enforced to zero. We employ 10-fold CV for each body-site data set: in each repetition, one-tenth of the samples are held out as a test set, the models are fitted to the remaining data, and predictions are generated for the held-out samples. This process is repeated ten times so that every sample serves as a test observation exactly once for each data set and taxon.

## Results

### Simulation

**Table 1:** MSE, denoted as $\overline{e^2}_{sp}$ with site $s=1,2$ and covariate $p=2,3$ for regression coefficients $\{\beta _{sp}\}$, with its standard error $\sigma _{\overline{e^2}_{sp}}$ in parentheses

Case	Model	$\overline{e^2}_{12} (\sigma _{\overline{e^2}_{12}}) \times 10^{5}$	$\overline{e^2}_{22} (\sigma _{\overline{e^2}_{22}}) \times 10^{5}$	$\overline{e^2}_{13} (\sigma _{\overline{e^2}_{13}}) \times 10^{2}$	$\overline{e^2}_{23} (\sigma _{\overline{e^2}_{23}}) \times 10^{2}$
$\phi ^2 = 0$	SNBM	2.21 (0.33)	1.23 (0.17)	3.63 (0.57)	1.11 (0.16)
	JNBM	2.40 (0.34)	1.19 (0.16)	3.70 (0.62)	1.20 (0.17)
	JNBM_SG	2.28 (0.33)	1.19 (0.16)	3.65 (0.61)	1.23 (0.17)
	JNBM_Mix	2.30 (0.33)	1.19 (0.16)	3.56 (0.61)	1.19 (0.17)
1	SNBM	5.81 (0.73)	3.72 (0.51)	11.85 (1.76)	6.76 (1.03)
	JNBM	3.65 (0.62)	2.01 (0.26)	6.68 (0.81)	5.43 (0.80)
	JNBM_SG	3.67 (0.57)	2.01 (0.26)	6.49 (0.82)	5.39 (0.76)
	JNBM_Mix	3.69 (0.58)	2.01 (0.26)	6.21 (0.80)	5.27 (0.73)
5	SNBM	23.28 (4.15)	30.29 (4.42)	27.92 (2.87)	38.13 (6.79)
	JNBM	9.14 (1.16)	7.06 (0.94)	13.81 (2.06)	10.30 (1.58)
	JNBM_SG	9.13 (1.14)	7.13 (0.97)	13.63 (2.05)	10.45 (1.62)
	JNBM_Mix	8.72 (1.04)	6.80 (0.87)	13.70 (2.02)	10.51 (1.64)
(1/2, 10)	SNBM	39.69 (6.55)	36.13 (5.20)	27.31 (2.74)	52.16 (8.98)
	JNBM	26.14 (4.36)	28.12 (5.03)	20.72 (2.44)	20.86 (2.60)
	JNBM_SG	6.86 (1.16)	5.61 (0.78)	8.67 (1.07)	7.51 (0.92)
	JNBM_Mix	16.13 (2.23)	15.49 (2.74)	14.78 (1.86)	14.84 (2.12)

We conduct simulation studies of paired samples under varying levels of association, comparing the separate negative binomial model (SNBM), the joint negative binomial model (JNBM), and its extensions JNBM_SG and JNBM_Mix. Synthetic data are generated by drawing $n=300$ pairs of samples from negative binomial distributions with latent factors defined as,

$$\begin{aligned} \begin{aligned} y_{1i}&\overset{\textit{ind.}}{\sim }\text {NB}(\exp (\gamma _i+\log (N_{1i})+X_i'\beta _1),\alpha _1);\\ y_{2i}&\overset{\textit{ind.}}{\sim }\text {NB}(\exp (\gamma _i+\log (N_{2i})+X_i'\beta _2),\alpha _2);\\ \gamma _i&\overset{\textit{i.i.d.}}{\sim }\text {N}(-\phi ^2/2,\phi ^2). \end{aligned} \end{aligned}$$

The dispersion parameters and regression coefficients are arbitrarily chosen to be $\alpha _1=2$, $\alpha _2=1$, $\beta _1=(\beta _{11},\beta _{12},\beta _{13})=(-10,0.03,0.03)$, and $\beta _2=(\beta _{21},\beta _{22},\beta _{23})=(-9,0.02,0.01)$, where $X_i=(x_{i1},x_{i2},x_{i3})'$ is a vector consisting of an intercept $x_{i1}$, a continuous covariate $x_{i2} \sim \text {Unif}(20,80)$ and a binary covariate $x_{i3} \sim \text {Bern}(0.3)$. $\text {Unif}(a,b)$ and $\text {Bern}(p)$ are (continuous) uniform and Bernoulli distributions with means $(a+b)/2$ and *p*, respectively.

Again, the dispersion parameter $\phi ^2$ of the latent factors in the underlying distribution determines the strength of association between paired samples. It is set to 0 for independent pairs and to 1 or 5 for dependent pairs, with 5 indicating stronger association. We also consider a scenario in which the association strength varies across samples, with 80% of samples randomly assigned $\phi ^2=10$ and the remainder assigned $\phi ^2=0.5$. $N_{1i}$ and $N_{2i}$ are offsets, which in real-world applications normalize for sequencing depth (or the sum of abundances across selected taxa of interest). We set $N_{1i} \overset{\textit{i.i.d.}}{\sim }\text {NB}(8000000,1)+8000000$ and $N_{2i} \overset{\textit{i.i.d.}}{\sim }\text {NB}(2000000,1)+2000000$; sampling $N_{si}$ from the negative binomial distribution produces sequencing depths with right skewness, as observed in the UMICRO data set. Using the parameters, covariates, and offsets defined above, we generated 100 sets of 300 sample pairs for analysis. Sparsity (the proportion of zero counts) varies across scenarios and reaches at most 20% on average in the final scenario with two different levels of paired association.

We evaluate the models with regard to the accuracy of their estimated regression coefficients. Table 1 shows the mean squared error (MSE) between the estimated and true coefficients for the two covariates under each model, demonstrating that JNBM is superior to SNBM for the data with non-zero associations ($\phi ^2 \ne 0$). This indicates that ignoring the latent sample effect $(\gamma _i)$, as in SNBM, can reduce efficiency in estimating the underlying relationships between the response variables and predictors. In other words, the loss of efficiency is an artifact of model misspecification: SNBM does not account for correlation between the two data sets, treating them as independent despite shared effects on their abundances. The gap in estimation accuracy between the two models widens as the paired association in the underlying distribution strengthens. Regression estimates from JNBM_SG and JNBM_Mix are comparable to those from SNBM and JNBM when no more than one paired association is present, but the extended models provide superior performance when two distinct association strengths exist across samples. JNBM_SG requires an additional covariate corresponding to the grouping variable *g*(*i*). For the (0.5, 10) case, we use the true allocation parameter from data generation to assign the dispersion parameters; for the other cases, $g(i) \in \{1,2\}$ is sampled randomly. For JNBM_Mix, we set $L=3$ for all cases. Under (0.5, 10), JNBM_SG attains lower MSEs than JNBM_Mix because it uses the true allocation, whereas JNBM_Mix must estimate $\xi _i \in \{1,2,3\}$.

Additionally, we compare JNBM with a Gaussian mixed model (GMM) using synthetic data, where both models include random effects $\gamma _i$. JNBM incorporates sequencing depth as an offset, whereas GMM normalizes counts by sequencing depth and applies a log transformation with an added pseudocount to map responses to the real line. Because no universally optimal pseudocount exists, we consider four choices: $10^{-4}$, $10^{-7}$, $10^{-10}$, and a data-dependent choice based on $(y_{si},N_{si})$, defined for each *s* as $\min \{0.5 \times y_{si}/N_{si}: y_{si} \ne 0, i = 1,\ldots ,n\}$. Pair data are generated from the Lomax distribution $\text {Lo}(2,\mu _{si})$, a heavy-tailed distribution with mean $\mu _{si} = \exp (\gamma _i + X_i'\beta _s)$ and infinite variance, which is therefore neither negative binomial nor Gaussian; counts are obtained by rounding the Lomax draws. The heavy-tailed property captures sparsity, with many zeros alongside substantial non-zero values. With fixed regression coefficients $\beta _1=(0,-3,1)$ and $\beta _2=(-3,-2,1.5)$, covariates are generated as $X_i = (x_{i1}, x_{i2}, x_{i3})'$, where $x_{i1}=1$, $x_{i2}\sim \text {N}(5,1)$, and $x_{i3}\sim \text {N}(0,1)$. Sequencing depths are simulated as $N_{1i} \overset{\textit{i.i.d.}}{\sim }\text {NB}(200000,1)+200000$ and $N_{2i} \overset{\textit{i.i.d.}}{\sim }\text {NB}(100000,1)+100000$, and random effects follow $\gamma _i \sim \text {N}(0,5)$. The proportion of zero counts, averaged across 100 iterations, is approximately 70% for $y_{1i}$ and 60% for $y_{2i}$, with sample sizes of 100 for each. Using these data, we benchmark JNBM against SNBM a second time to assess whether its superiority persists under non-NB underlying distributions.

Table 2 illustrates a key limitation of GMM: its high sensitivity to the choice of pseudocount. We did not identify a single value that achieved the most accurate estimates across all coefficients. For this data set, a pseudocount of $10^{-7}$ resulted in smaller MSEs for $\beta _{22}$ and $\beta _{23}$, whereas $10^{-10}$ provided better performance for $\beta _{12}$ and $\beta _{13}$. The data-driven choice $\min (y_{si}/N_{si})$ can serve as a general option, but it does not guarantee optimal MSEs. In contrast, JNBM directly models the count data and thus avoids this sensitivity. It shows superior performance to SNBM in the presence of a non-zero paired association, even when the data are generated from a non-NB distribution.

**Table 2:** MSE (SE) of the regression coefficients ${\beta _{sp}}$, with $s=1,2$ and $p=2,3$, under GMM with four different pseudocount (PC) choices, and under SNBM and JNBM

Model	$\overline{e^2}_{12} (\sigma _{\overline{e^2}_{12}})$	$\overline{e^2}_{22} (\sigma _{\overline{e^2}_{22}})$	$\overline{e^2}_{13} (\sigma _{\overline{e^2}_{13}})$	$\overline{e^2}_{23} (\sigma _{\overline{e^2}_{23}})$
GMM ($\min (y_{si}/N_{si})$ PC)	3.63 (0.09)	1.07 (0.04)	0.47 (0.02)	0.71 (0.03)
GMM ($10^{-4}$ PC)	7.36 (0.05)	2.85 (0.03)	0.91 (0.01)	1.80 (0.02)
GMM ($10^{-7}$ PC)	2.19 (0.08)	0.31 (0.03)	0.30 (0.02)	0.23 (0.02)
GMM ($10^{-10}$ PC)	0.24 (0.03)	1.12 (0.09)	0.16 (0.02)	0.63 (0.06)
SNBM	0.35 (0.03)	0.09 (0.01)	0.29 (0.05)	0.27 (0.04)
JNBM	0.17 (0.02)	0.07 (0.01)	0.12 (0.02)	0.11 (0.02)

Table 3 provides additional comparative results across a broader range of simulation settings, including smaller sample sizes ($n = 50$ for both $s=1,2$), larger sequencing depths ($N_{1i} \overset{\textit{i.i.d.}}{\sim }\text {NB}(800000, 1) + 800000$ and $N_{2i} \overset{\textit{i.i.d.}}{\sim }\text {NB}(300000, 1) + 300000$), smaller sequencing depths ($N_{1i} \overset{\textit{i.i.d.}}{\sim }\text {NB}(70000, 1) + 70000$ and $N_{2i} \overset{\textit{i.i.d.}}{\sim }\text {NB}(40000, 1) + 40000$), and alternative sampling distributions (rounded exponential samples and Poisson counts).

When the sequencing depth increases, the mean proportion of zero counts across 100 repetitions decreases to approximately 60% and 50% for $y_{1i}$ and $y_{2i}$, respectively. In contrast, the lower sequencing depth increases these proportions to about 80% and 70%. The two alternative sampling distributions represent cases with reduced sparsity: the Poisson distribution can be viewed as a special case of the negative binomial distribution with overdispersion parameter $\alpha = 0$, where the variance equals the mean. The exponential distribution has an exponential tail and finite variance, whereas the Lomax distribution is heavy-tailed, yielding infinite variance when the shape parameter is set to 2.

In each scenario, all other simulation settings remained unchanged. These analyses demonstrate that the sensitivity of the GMM to pseudocounts (as shown in Table 2) is not an artifact of that discretized Lomax setting, further revealing the inherent difficulty of selecting an appropriate pseudocount, since no universal choice exists that ensures optimal performance across conditions. The examples also illustrate the JNBM’s competitive performance in coefficient estimation. The GMM’s results may be influenced by bias introduced through pseudocount adjustments and by its assumption of unimodality at a positive value, whereas the synthetic data have a mode at zero.

**Table 3:** MSE (SE) of the regression coefficients ${\beta _{sp}}$, with $s=1,2$ and $p=2,3$. GMM1: pseudocount = $\min (y_{si}/N_{si})$; GMM2: pseudocount = $10^{-10}$

Case	Model	$\overline{e^2}_{12} (\sigma _{\overline{e^2}_{12}})$	$\overline{e^2}_{22} (\sigma _{\overline{e^2}_{22}})$	$\overline{e^2}_{13} (\sigma _{\overline{e^2}_{13}})$	$\overline{e^2}_{23} (\sigma _{\overline{e^2}_{23}})$
Smaller *n*	GMM1	3.32 (0.09)	0.82 (0.04)	0.53 (0.03)	0.80 (0.04)
	GMM2	0.38 (0.05)	1.25 (0.12)	0.43 (0.06)	0.89 (0.10)
	JNBM	0.26 (0.03)	0.16 (0.04)	0.50 (0.09)	0.30 (0.05)
Larger $N_{si}$	GMM1	2.34 (0.07)	0.64 (0.03)	0.32 (0.02)	0.46 (0.03)
	GMM2	0.43 (0.05)	1.24 (0.10)	0.18 (0.03)	0.76 (0.07)
	JNBM	0.19 (0.02)	0.08 (0.01)	0.10 (0.01)	0.12 (0.02)
Smaller $N_{si}$	GMM1	4.75 (0.09)	1.45 (0.04)	0.61 (0.02)	0.97 (0.03)
	GMM2	0.27 (0.05)	0.82 (0.09)	0.18 (0.03)	0.44 (0.05)
	JNBM	0.14 (0.02)	0.07 (0.01)	0.31 (0.07)	0.19 (0.03)
Exponential	GMM1	3.29 (0.07)	0.88 (0.04)	0.43 (0.02)	0.60 (0.03)
	GMM2	0.35 (0.04)	1.38 (0.11)	0.23 (0.03)	0.86 (0.10)
	JNBM	0.12 (0.01)	0.07 (0.01)	0.23 (0.11)	0.22 (0.11)
Poisson	GMM1	2.93 (0.07)	0.73 (0.03)	0.39 (0.02)	0.50 (0.02)
	GMM2	0.61 (0.06)	1.73 (0.12)	0.24 (0.04)	0.98 (0.09)
	JNBM	0.08 (0.01)	0.04 (0.01)	0.22 (0.03)	0.15 (0.02)

### Case study: the UMICRO Data

We apply JNBM to the UMICRO data set and compare the resulting inferences to those from SNBM in the estimation of regression coefficients and in the prediction of taxa abundance at one of the sites. Again, data were collected from two different body sites by catheterization for urine samples and vaginal swabbing for vaginal samples. Full-length contigs received from Loop Genomics (Element Biosciences, San Diego, CA), which we refer to as LoopSeq, were processed with DADA2 (v 1.24.0) to generate amplicon sequence variants (ASV), using parameters as recommended for synthetic full-length 16 S LoopSeq data in [21]. Taxonomic classifications were assigned using BLCA (v 2.2) and the 16 S NCBI database (downloaded on 11/16/2021). The processed data contain 68 sample pairs with 151 common taxa (genera) found in both vaginal and urine samples, while the following analysis focuses on nine taxa of interest: *Gardnerella, Streptococcus, Lactobacillus, Aerococcus, Anaerococcus, Bifidobacterium, Corynebacterium, Fannyhessea, Prevotella*. A boxplot of the sequencing depth distributions is provided in Figure S17 of Supplementary Material A.7. We incorporate several clinical and demographic metadata for the subjects as covariates in the models: age, body mass index (BMI), diabetes (yes/no), daily yogurt or probiotic consumption (yes/no), race_ethnicity (0: White, 1: Black, 2: Others), the presence or absence of overactive bladder (OAB) (yes/no). In addition, the study group variable on menopausal status (Postmenopausal with no estrogen; Postmenopausal on estrogen; Premenopausal) is used to cluster the samples for JNBM_SG, introduced in Section 2.2.

**Fig. 1:**
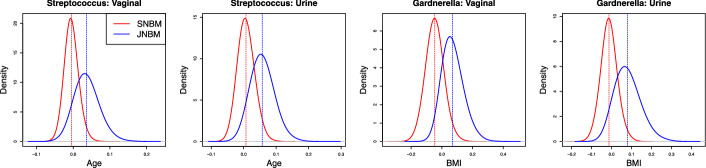
Posterior distributions of regression coefficients for age in the vaginal and urine data sets for *Streptococcus* (first two panels); and regression coefficients for BMI in the data sets for *Gardnerella* (last two panels). The dashed lines indicate the posterior means

Figure 1 shows examples of differences between SNBM and JNBM when it comes to the estimation of regression coefficients. JNBM indicates relatively pronounced positive trends of age and BMI with *Streptococcus* and *Gardnerella* counts in both vaginal and urine samples, while these trends are negligible under SNBM. In the previous section, we saw that the joint model provides more accurate estimation results when paired associations are present. Here, the posterior means (and standard deviations) of $\phi ^2$ for *Streptococcus* and *Gardnerella* are given by 16 (4) and 17 (5), respectively. It favors the results of JNBM for the regression coefficients. Furthermore, the positive relationships of age and BMI with the taxa under JNBM are consistent with the biological findings reported in [22, 23]. More results on regression coefficients can be found in Supplementary Material A.1.

**Fig. 2:**
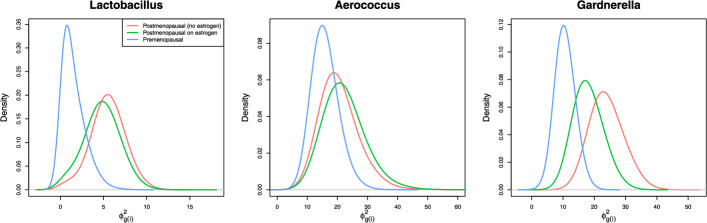
Posterior distributions of $\phi ^2_{g(i)}$, the dispersion hyperparameters of latent factors, from JNBM_SG for the three different genera

JNBM_SG clusters the samples according to the study group variable and allows each group to have its own distinct level of paired association. The first two panels of Fig. 2 illustrate that the two postmenopausal groups have similar association strengths but differ from the premenopausal group, which exhibits a weaker association. In the last panel, *Gardnerella* shows notable differences in association strength across the three groups, with the postmenopausal group without estrogen displaying the strongest association, followed by the postmenopausal group with estrogen. Figure S11 presents the posterior distributions of the parameters for all nine taxa.

**Fig. 3:**
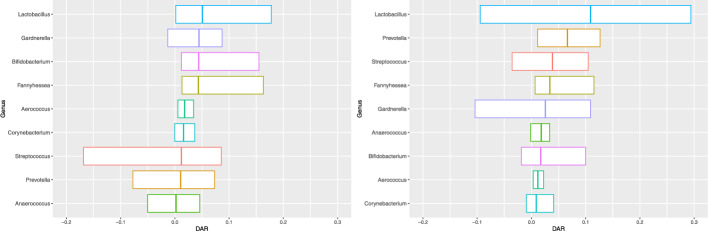
Boxplots (from the 1st to 3rd quartiles) of $\text {DAR}_{sik}^{\text {SNBM-JNBM}}\_\text {SG}$, the differences in the absolute residuals between SNBM and JNBM_SG, for each taxon. The genera on the y-axis are ordered by their median DARs in each data set, with vaginal on the left and urine on the right

To assess the predictive performance of the models for the microbial composition, we calculated the residuals between the observed and predicted relative abundances of each microbial genus, denoted as $r_{sik}^M = \frac{y_{sik}}{\sum _{k=1}^9 y_{sik}} - \frac{\tilde{y}_{sik}^M}{\sum _{k=1}^9 \tilde{y}_{sik}^M}$, where $y_{sik}$ is an observed count for the *k*-th taxon in the *i*-th sample of the *s*-th body site and $\tilde{y}_{sik}^M$ the corresponding predicted count under model *M*. Figure 3 presents the difference in the absolute residuals between SNBM and JNBM_SG, that is, $\text {DAR}_{sik}^\text {SNBM-JNBM\_SG} = |r_{sik}^\text {SNBM}|-|r_{sik}^\text {JNBM\_SG}|$. The greater the difference, the better the predictive performance of JNBM_SG. The boxplots represent the distributions of DARs per taxon for each body site, with the positive medians (in most taxa) indicating that JNBM_SG enhances the predictive efficiency of SNBM. The improvement in prediction under the joint model is attributed to the borrowing of information between sites. As such, the base joint model, JNBM, also outperforms SNBM, which has been further improved by accommodating different levels of association for clusters in JNBM_SG and JNBM_Mix (Figure S10).

As another measure of predictive performance, we computed Kullback–Leibler (K-L) divergence–type residuals, defined as $D^M_{si} = \sum _{k=1}^K p_{sik}\log (p_{sik}/\tilde{p}^M_{sik})$, where $p_{sik} = y_{sik}/\sum _{k=1}^K y_{sik}$ and $\tilde{p}^M_{sik} = \tilde{y}^M_{sik}/\sum _{k=1}^K \tilde{y}^M_{sik}$, with $K=9$. Again, $\tilde{y}^M_{sik}$ is a predicted value obtained from the posterior predictive samples (see prediction details in Section 2.3.2), not the fitted mean used in the deviance residuals. $\tilde{y}^M_{sik}$ can be zero when the recalculated sequencing depth for a test-set sample is zero, in which case the predicted value becomes zero across all NB-based models considered in this study. To avoid this issue, we recommend adding a small positive constant (we used 0.1) to the predicted values before computing $D^M_{si}$, which does not affect the relative ranking of predictive performance across models. The means across samples $(\bar{D}^M_{s} = \sum _{i=1}^n D^M_{si}/n)$ for SNBM and JNBM_SG are 4.45 and 3.09, respectively, for vaginal data, and 3.60 and 2.62, respectively, for urine data. These results are consistent with the superior predictive performance of JNBM_SG observed in the DAR above. Distributions of $D^M_{si}$ for $s=1,2$ under each model (*M*) are presented as boxplots in Figure S13 of Supplementary Material A.5. In addition, to examine whether the difference between $N_{1i}$ and $N_{2i}$ influences inference for a given sample, we tested the association between the absolute differences and the residuals $D^M_{si}$ using cor.test() in R and found no significant correlations across all sites *s* and models *M*.

Lastly, regarding computational intensity for posterior inference, each taxon needs a different burn-in period until the posterior distributions of model parameters stabilize. Empirically, 1,000,000 MCMC iterations with a burn-in of 500,000 achieves convergence of the posterior distributions for almost all taxa across models. On an Apple M1 machine (8-core CPU, 8 GB RAM), posterior inference takes approximately 1.5 h for SNBM, 2 h for JNBM and JNBM_SG, and 3.5 h for JNBM_Mix with $L=8$.

## Conclusions

We have proposed a joint negative binomial model (JNBM), a latent factor model based on negative binomial distributions, for paired microbiome data. The UMICRO data, gathered from two different body sites for each of the subjects, is a motivating real-world example, and the proposed model incorporates a set of latent factors to capture associations between the two body sites. JNBM has been extended by modifying the distribution for the latent factors, which permits varying levels of paired association across groups of samples.

We focus on paired data in this paper. Extensions to more than two sites ($s=1,\ldots ,S$, $S>2$) are outside the scope of this work, though the latent factor model can be generalized in such cases. Specifically, the single latent factor $\gamma _i$ may be replaced with $\boldsymbol{\gamma }_i = (\gamma _{i1},\ldots ,\gamma _{iJ})'$, $\gamma _{ij} \overset{\textit{ind.}}{\sim }\text {N}(-\phi ^2_j/2,\phi ^2_j)$, yielding $\mu _{si} = \big (\boldsymbol{\lambda }_s'\boldsymbol{\gamma }_i+\log (N_{si})+X_i'\beta _{s}\big )$, where $\boldsymbol{\lambda }_s = (\lambda _{s1},\ldots ,\lambda _{sJ})'$ and $J \ll S$. For identifiability, one factor loading vector may be fixed (e.g., $\boldsymbol{\lambda }_1 = (1,\ldots ,1)'$), while priors such as normal or horseshoe distributions can be assigned to the others. The latter encourages shrinkage, allowing many loadings to be close to zero so that only a subset remain active. The model reduces to JNBM when $J=1$ and $\lambda _s=1$. Allowing $\lambda _2$ to vary may capture negative paired associations, but we restrict attention to positive associations, consistent with our hypothesis that the proximity of vaginal and urine sites promotes similar microbial compositions.

The cross-site prediction can serve as an effective means of checking model adequacy. A persistent and large discrepancy between the predicted and observed counts may suggest that the single–latent-factor specification is inadequate, and further extensions with additional latent factors may be required. It can also indicate that the samples are highly heterogeneous and that unstructured variability not accounted for in our latent factor model may be prevalent. A third, hopefully less frequent, possibility is that there are systematic biases or data issues at one of the sites. This may prompt the practitioner to examine the sampling and preprocessing steps.

Our joint negative binomial model (JNBM) provides more accurate regression estimates than the separate negative binomial model (SNBM) when there exists a non-zero paired association in the data. The extended models (JNBM_SG and JNBM_Mix) provide regression estimates comparable to those from SNBM and JNBM when there are zero or only a single paired association, but they perform better when two different association strengths exist across samples. A comparison of JNBM with a Gaussian mixed model (GMM) highlights the limitation of GMM, its high sensitivity to the pseudocounts required for transforming counts into real values. Moreover, JNBM continues to outperform SNBM when the data are generated from a non-NB underlying distribution with nonzero paired associations.

In the UMICRO case study, the joint model reveals conspicuous positive patterns of BMI and age with *Gardnerella* and *Streptococcus* abundances, respectively, which are in agreement with some previous research findings [22, 23]. Moreover, the joint model outperforms the separate model in prediction, which can be attributed to the use of observations from the opposite body site for prediction via latent factors. With extended models, prediction performance can be further enhanced due to their ability to assign different association strengths to each group. In addition, estimates of the association strengths can also be used to characterize sample groups. For example, in the UMICRO case study, we found that the two postmenopausal cohorts (with and without estrogen) exhibited similar associations, whereas the premenopausal cohort showed relatively weaker associations for *Lactobacillus* and *Aerococcus*. On the other hand, *Gardnerella* displayed differences across all cohorts.

Although the case study focuses on nine taxa of interest, we have applied these models to other genera. Unsurprisingly, the models struggled to predict taxa that are prevalent in one site but rare in the other. For example, *Escherichia*, *Klebsiella*, and *Pseudomonas* are pathogens that are common in the urine but scarce in the vagina, showing a high sparsity (high proportion of samples with zero count) in the vaginal data set. It is particularly challenging to predict non-zero counts (urine) using zero counts (vaginal) compared to the reverse scenario. The shared latent factor assumption under our model also becomes questionable when the taxon is rarely present in one of the sites but common in the other.

Finally, the predictive performance is evaluated using relative abundances, as microbial composition is of primary interest, by comparing predicted values with observed ones. Previous work [24] has questioned the validity of measured relative abundances, noting that they do not represent the true (ground-truth) composition but rather the product of the actual abundances and relative efficiencies (taxon- and protocol step–specific biases). Because our analysis does not account for such biases in microbial composition, we acknowledge that the predicted relative abundances may not reflect the true underlying composition.

## Supplementary Information

Supplementary Material 1

## Data Availability

Raw sequencing files and associated metadata will be publicly shared on the Sequence Read Archive (SRA), Bioproject Accession Number: PRJNA685466. The UMICRO data used for the case study were generated using the sequencing files, with a description of the data generation provided in Section 3.2. To Additional information about the UMICRO data is available at: https://github.com/KarstensLab?tab=repositories. The R code implementing our model and analyses is available on GitHub at https://github.com/HyotaeKim/JNBM.
