# Supplementary material for: A negative binomial latent factor model for paired microbiome sequencing data
Source: BMC Bioinformatics. 2026 Jan 22;27:45. doi: 10.1186/s12859-025-06362-3 (PMC12910815; doi:10.1186/s12859-025-06362-3)
Supplement: Supplementary file 1 — Supplementary Material 1 [file 12859_2025_6362_MOESM1_ESM.pdf]

Supplementary to "A negative binomial latent factor model for paired microbiome sequencing data"

Hyotae Kim[1], Nazema Y. Siddiqui[2], Lisa Karstens[3], and Li Ma[4]

[1]Department of Biostatistics & Bioinformatics, Duke University
[2]Department of Obstetrics and Gynecology, Duke University
[3]Department of Medical Informatics and Clinical Epidemiology and Department of Obstetrics and Gynecology, Oregon Health & Science University
[4]Department of Statistics and Data Science Institute, University of Chicago

A. Additional figures for real data analysis

A.1 Posterior distributions of β_s

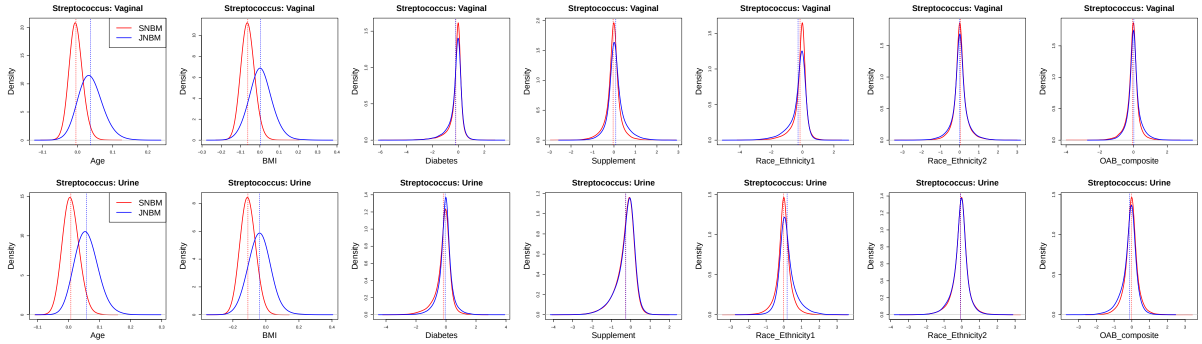

Figure S1: Posterior distributions of regression coefficients in the vaginal (first row) and urine (second row) data sets for *Streptococcus*.

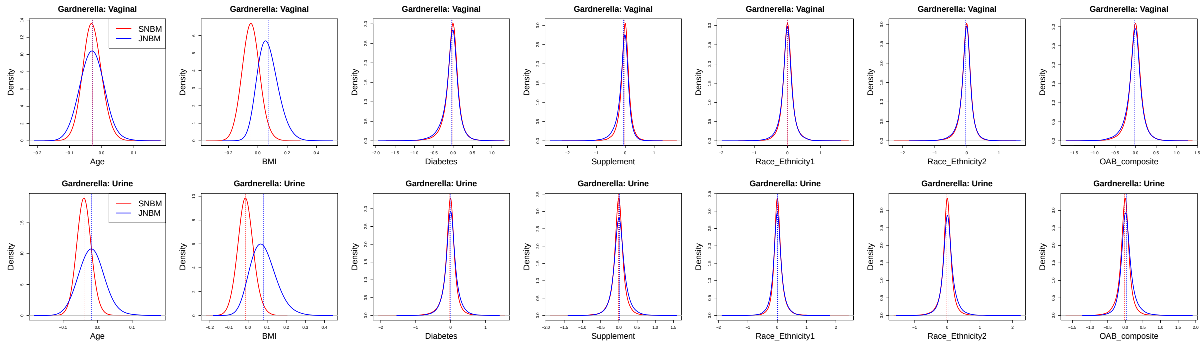

Figure S2: Posterior distributions of regression coefficients in the vaginal (first row) and urine (second row) data sets for *Gardnerella*.

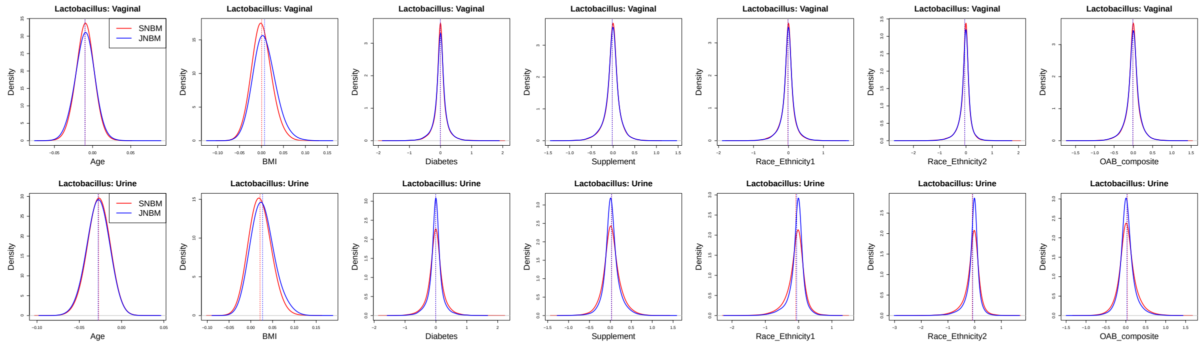

Figure S3: Posterior distributions of regression coefficients in the vaginal (first row) and urine (second row) data sets for *Lactobacillus*.

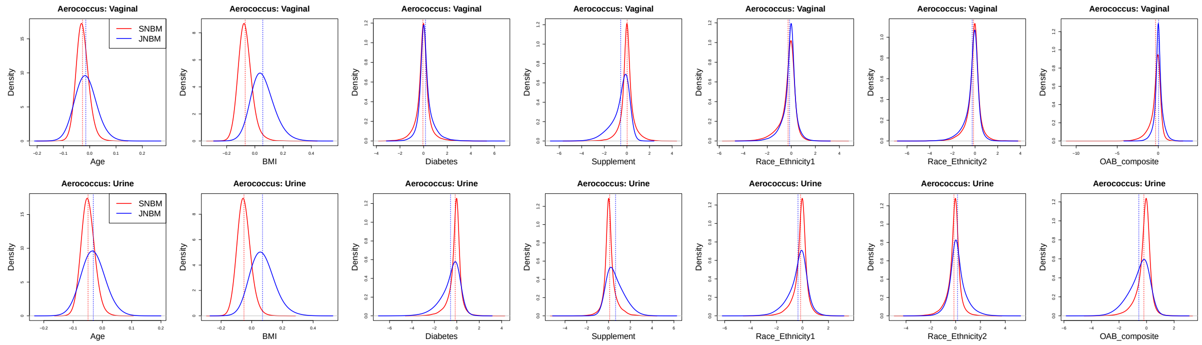

Figure S4: Posterior distributions of regression coefficients in the vaginal (first row) and urine (second row) data sets for *Aerococcus*.

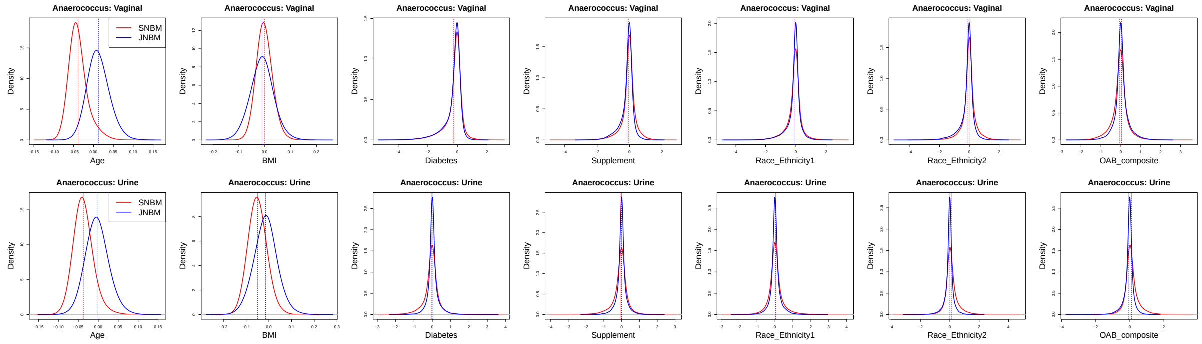

Figure S5: Posterior distributions of regression coefficients in the vaginal (first row) and urine (second row) data sets for *Anaerococcus*.

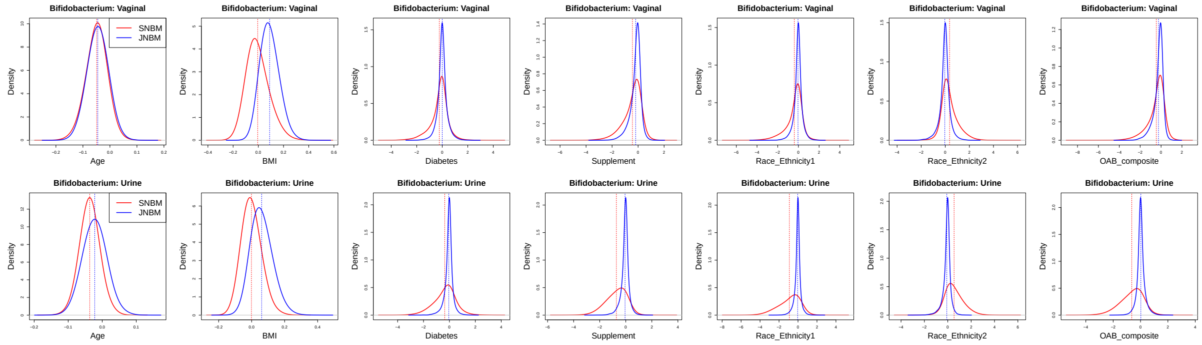

Figure S6: Posterior distributions of regression coefficients in the vaginal (first row) and urine (second row) data sets for *Bifidobacterium*.

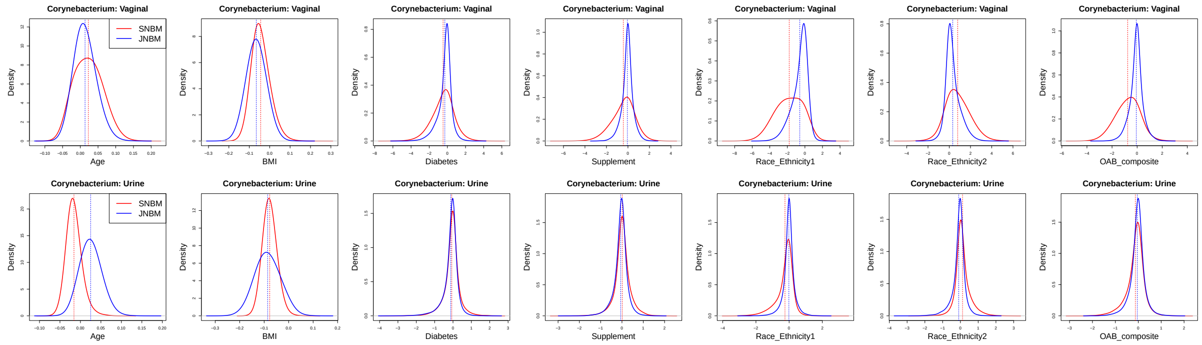

Figure S7: Posterior distributions of regression coefficients in the vaginal (first row) and urine (second row) data sets for *Corynebacterium*.

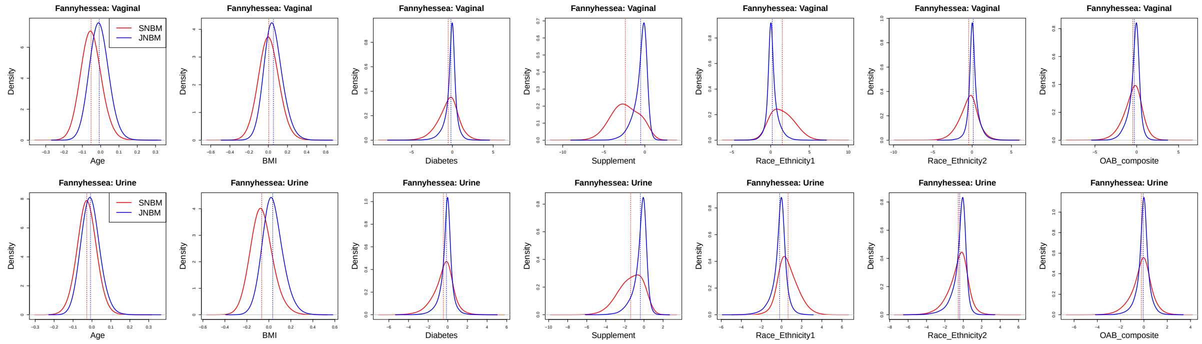

Figure S8: Posterior distributions of regression coefficients in the vaginal (first row) and urine (second row) data sets for *Fannyhessea*.

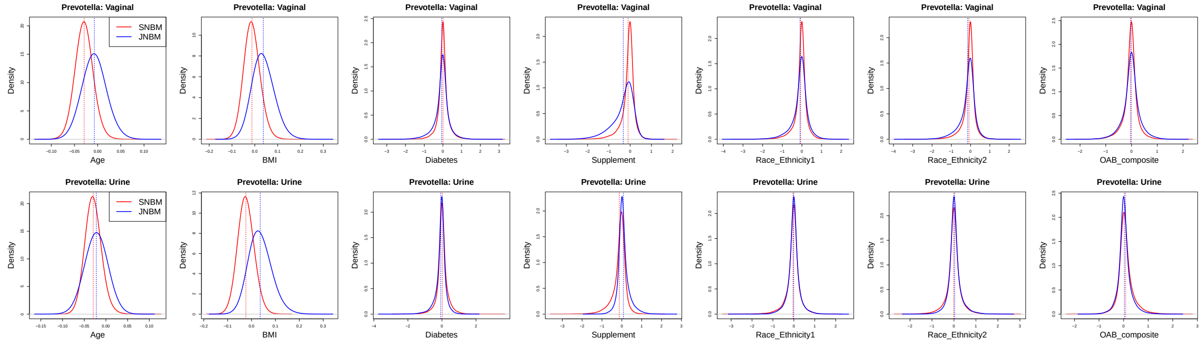

Figure S9: Posterior distributions of regression coefficients in the vaginal (first row) and urine (second row) data sets for *Prevotella*.

A.2 Boxplots of DARs

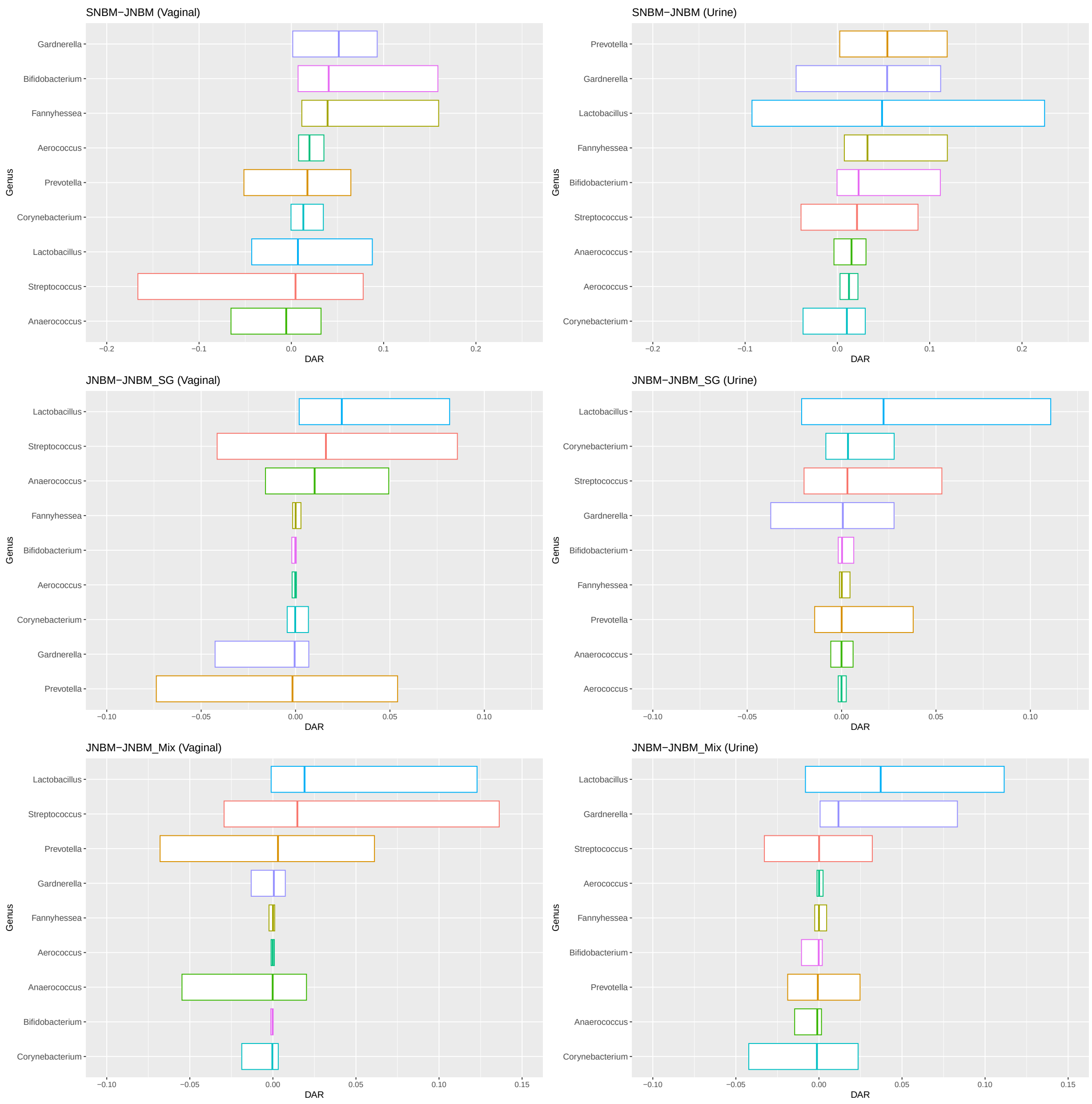

Figure S10: Boxplots of $\mathrm{DAR}_{sik}^{\text{SNBM-JNBM}}$ (top row), $\mathrm{DAR}_{sik}^{\text{JNBM-JNBM\_SG}}$ (middle row), and $\mathrm{DAR}_{sik}^{\text{JNBM-JNBM\_Mix}}$ (bottom row).

A.3 Posterior distributions of $\phi^2_{g(i)}$ under JNBM_SG

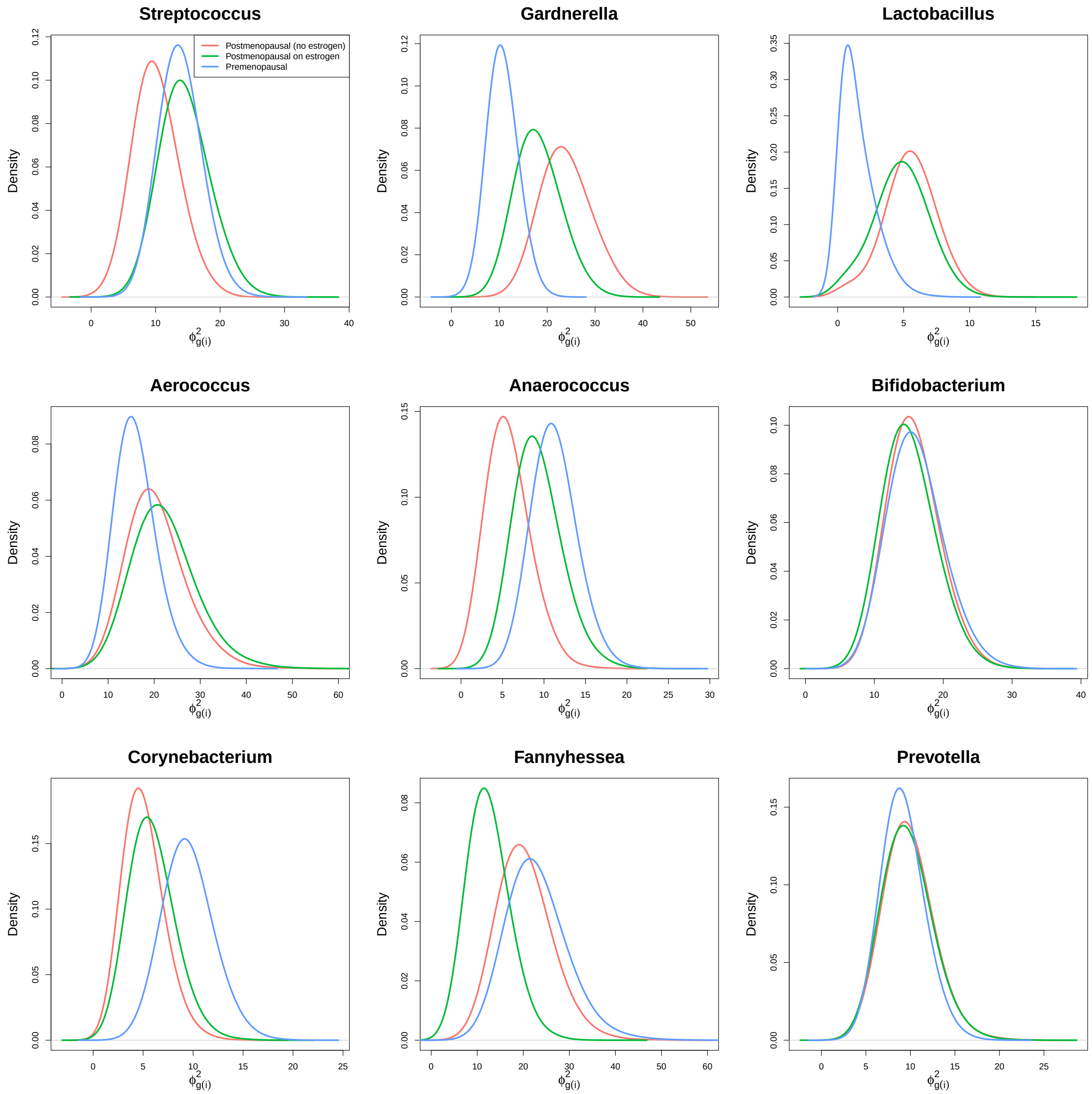

Figure S11: Posterior distributions of $\phi^2_{g(i)}$ colored by Study Group $g(i)$.

A.4 Prediction results of JNBM_Mix

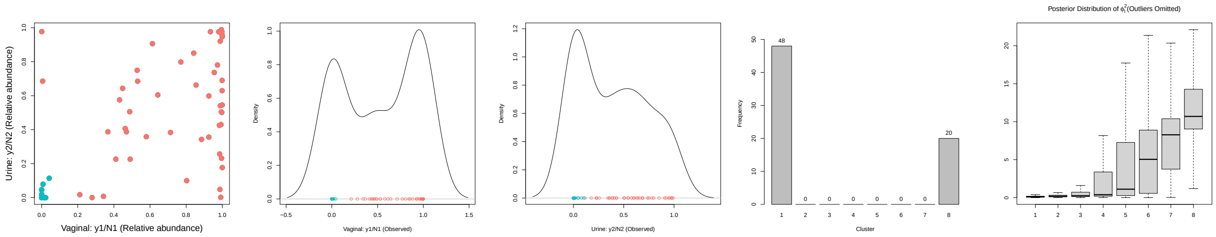

Figure S12: *Lactobacillus.* The left panel shows a scatterplot of observed relative abundances, colored by posterior estimates of the auxiliary variable ξ_i (red: $\hat{\xi}_i = 1$; green: $\hat{\xi}_i = 8$), where $\hat{\xi}_i$ is the posterior mode estimate. The next two panels display marginal density estimates of the relative abundances, with observed values shown at the bottom. The fourth panel is a barplot of ξ_i, and the final panel presents boxplots of the posterior distributions of the eight dispersion hyperparameters ϕ_l^2 for $l = 1, \ldots, 8$.

Taxon	$L = 4$	$L = 8$
Streptococcus	2	2
Gardnerella	2	2
Lactobacillus	2	2
Aerococcus	2	2
Anaerococcus	4	6
Bifidobacterium	4	7
Corynebacterium	3	4
Fannyhessea	2	2
Prevotella	3	5

Table S1: Estimated number of clusters for $L = 4$ and $L = 8$.

A.5 Boxplots of Kullback–Leibler divergence–type residuals

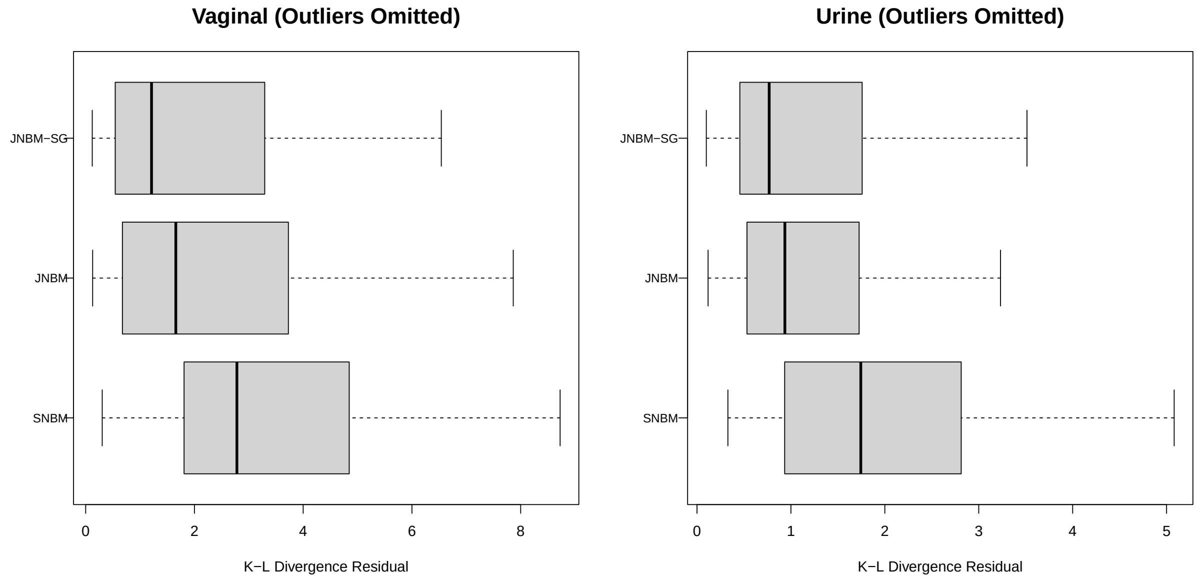

Figure S13: Boxplots of D_{si}^{M} for vaginal ($s = 1$, left) and urine ($s = 2$, right) data under each model M.

A.6 Sensitivity analysis of JNBM for *Streptococcus*

A.6.1 Sensitivity to a_α

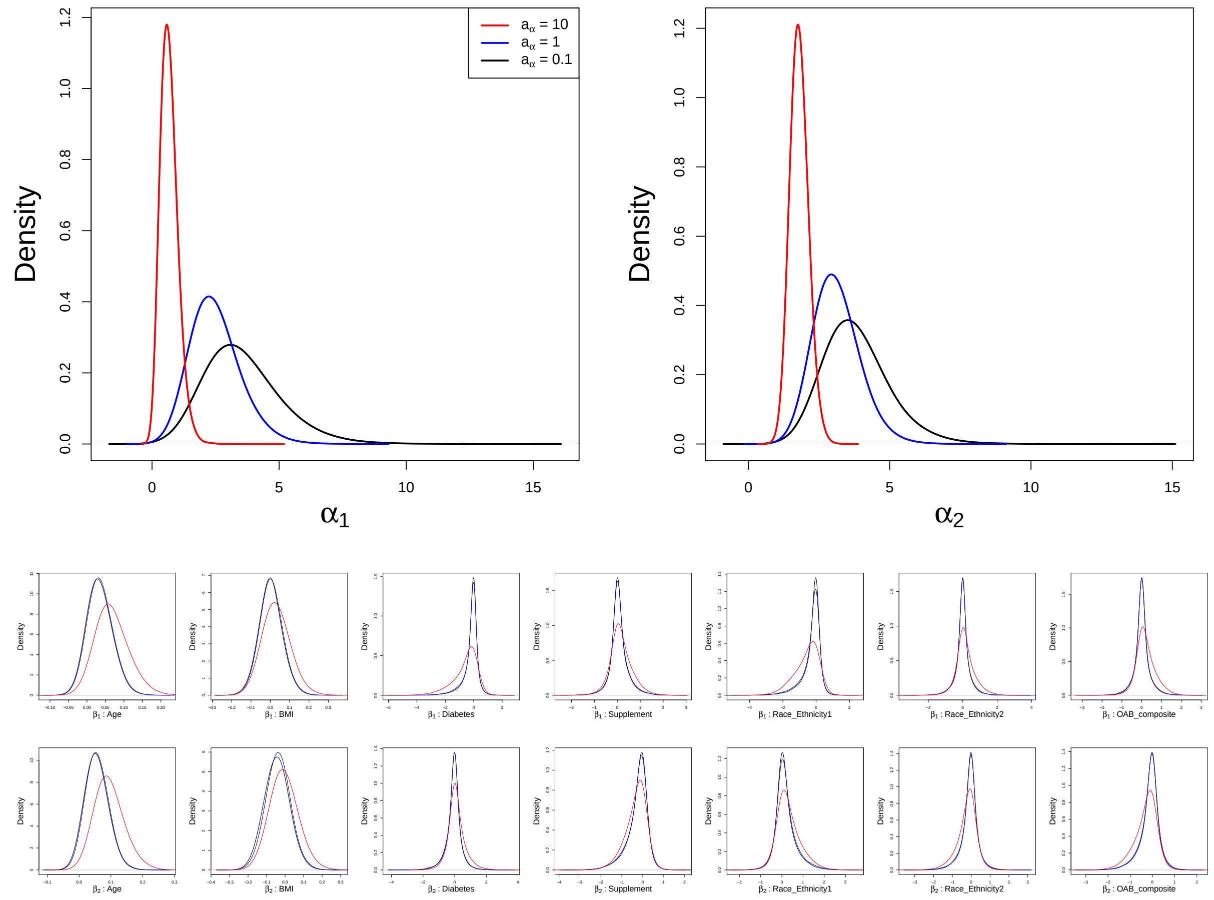

Figure S14: Posterior distributions of α_1, α_2, β_1, and β_2 under different choices of a_α.

A.6.2 Sensitivity to a_{ϕ^2}

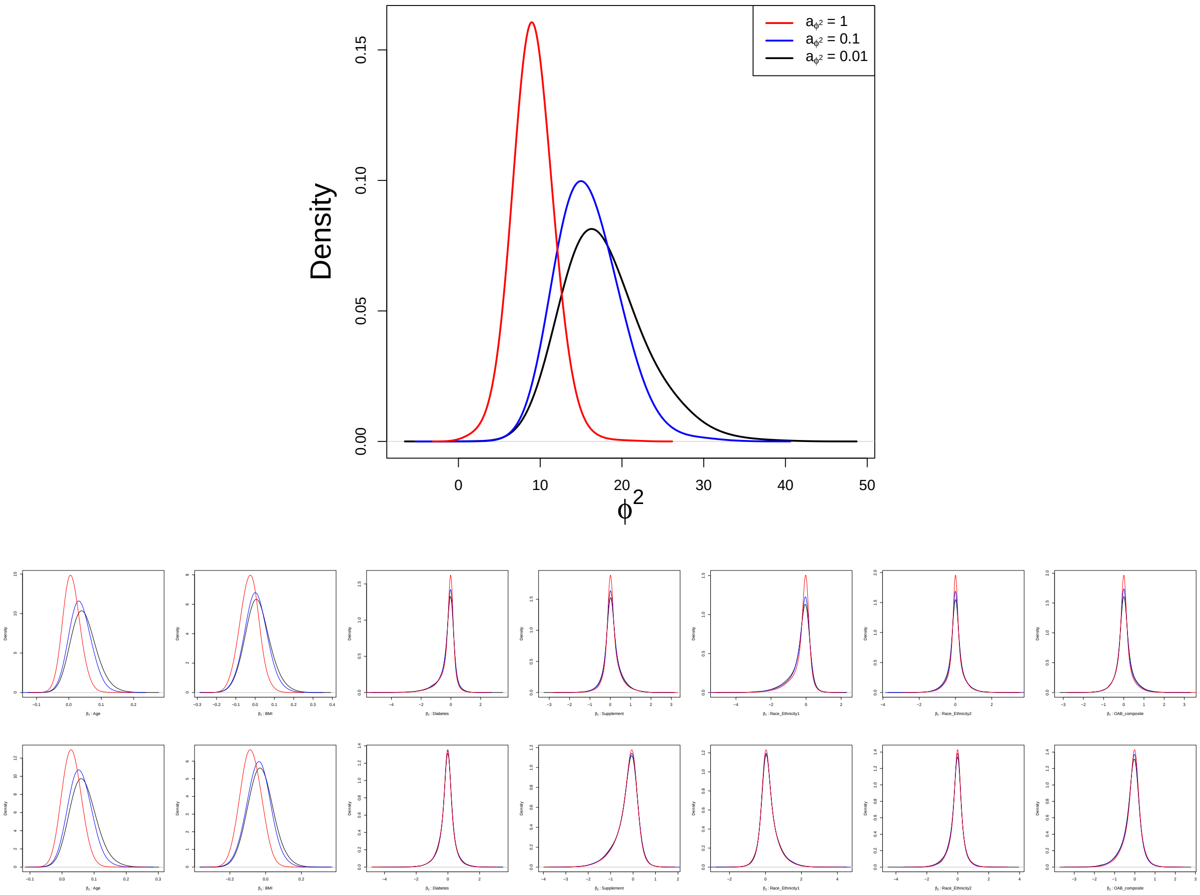

Figure S15: Posterior distributions of ϕ^2, β_1, and β_2 under different choices of a_{ϕ^2}.

A.6.3 Sensitivity to a_{τ^2}

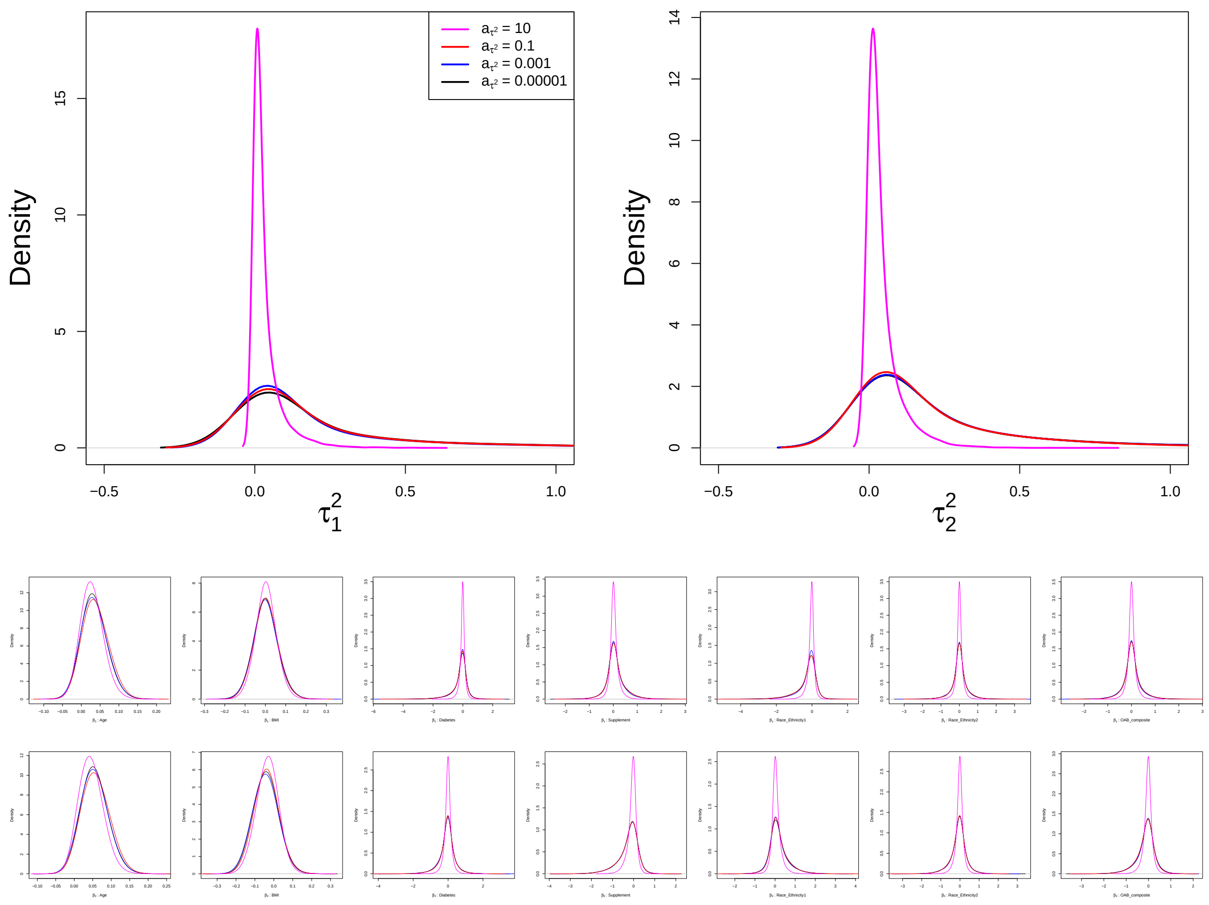

Figure S16: Posterior distributions of τ_1^2, τ_2^2, β_1, and β_2 under different choices of a_{τ^2}.

A.7 Boxplot of distributions of N_{1i} and N_{2i}

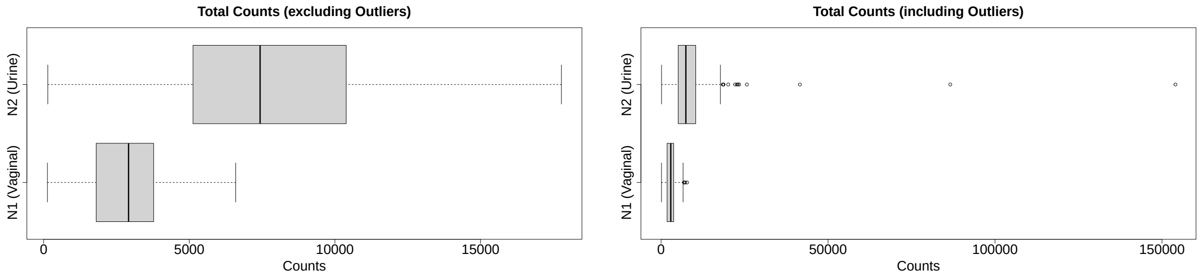

Figure S17: Boxplots of distributions of N_{1i} and N_{2i}.

B. Computational details on posterior inference

B.1 Pólya-Gamma augmentation for negative binomial models

This section describes the Pólya Gamma augmentation scheme for the proposed negative binomial model. For sample i at body site s, the negative binomial probability mass function is given by

$$\begin{aligned} p(y_{si}|\alpha_s, \beta_s, \gamma_i) &= \frac{\Gamma(y_{si} + \alpha_s^{-1})}{\Gamma(y_{si} + 1)\Gamma(\alpha_s^{-1})} \Big(\frac{\alpha_s^{-1}}{\alpha_s^{-1} + \mu_{si}}\Big)^{\alpha_s^{-1}} \Big(\frac{\mu_{si}}{\alpha_s^{-1} + \mu_{si}}\Big)^{y_{si}} \\ &= \frac{\Gamma(y_{si} + \alpha_s^{-1})}{\Gamma(y_{si} + 1)\Gamma(\alpha_s^{-1})} (\alpha_s^{-1})^{\alpha_s^{-1}} \Big[\frac{\mu_{si}^{y_{si}}}{(\alpha_s^{-1} + \mu_{si})^{\alpha_s^{-1} + y_{si}}}\Big] \\ &= \frac{\Gamma(y_{si} + \alpha_s^{-1})}{\Gamma(y_{si} + 1)\Gamma(\alpha_s^{-1})} \Big[\frac{(\mu_{si}\alpha_s)^{y_{si}}}{(1 + \mu_{si}\alpha_s)^{\alpha_s^{-1} + y_{si}}}\Big], \end{aligned}$$

where $\mu_{si} = \exp(\gamma_i + \log(N_{si}) + X_i'\beta_s)$ under JNBM. Following Polson *et al.* (2013), the last term in the square brackets can be expressed as,

$$\begin{aligned} \frac{(\mu_{si}\alpha_s)^{y_{si}}}{(1 + \mu_{si}\alpha_s)^{\alpha_s^{-1} + y_{si}}} &= \frac{(\exp(Z_{si}))^{y_{si}}}{\Big(1 + \exp(Z_{si})\Big)^{\alpha_s^{-1} + y_{si}}} \\ &= 2^{-(\alpha_s^{-1} + y_{si})} \exp\Big(Z_{si}(y_{si} - (\alpha_s^{-1} + y_{si})/2)\Big) \\ &\times \int_0^\infty \exp\Big(-\omega_{si} Z_{si}^2/2\Big) \mathrm{PG}(\omega_{si}|\alpha_s^{-1} + y_{si}, 0) d\omega_{si}, \end{aligned} \tag{1}$$

where $Z_{si} \equiv \log(\mu_{si}) + \log(\alpha_s)$. $\mathrm{PG}(\omega|a, b)$ denotes the probability density function of the Pólya-Gamma distribution $\mathrm{PG}(a, b)$. Using the (Pólya-Gamma) auxiliary variable ω_{si}, the equation (1) can be represented hierarchically without the integration. Then, the joint probability function for y_{si} and ω_{si} is derived as,

$$\begin{aligned} p(y_{si}, \omega_{si}|\alpha_s, \beta_s, \gamma_i) = \frac{\Gamma(y_{si} + \alpha_s^{-1})}{\Gamma(y_{si} + 1)\Gamma(\alpha_s^{-1})} 2^{-(\alpha_s^{-1} + y_{si})} \exp\Big(Z_{si}(y_{si} - (\alpha_s^{-1} + y_{si})/2)\Big) \\ \times \exp\Big(-\omega_{si} Z_{si}^2/2\Big) \mathrm{PG}(\omega_{si}|\alpha_s^{-1} + y_{si}, 0). \end{aligned}$$

The augmented likelihood with the Pólya-Gamma auxiliary variables ensures (normal) prior conjugacy for (β_s, γ_i), facilitating posterior inference, which will be discussed in the following section.

B.2 Posterior simulation

The Gibbs sampler is used to draw posterior samples of model parameters for inference. The Pólya-Gamma augmentation enables us to obtain the full conditionals in closed form for most of JNBM parameters except $\boldsymbol{\alpha} = \{\alpha_1, \alpha_2\}$, ϕ^2, and $\boldsymbol{\tau^2} = \{\tau_1^2, \tau_2^2\}$, for which we employ the Metropolis–Hastings algorithm. The following are the full conditionals for the parameters of our joint models.

Let $\boldsymbol{y_{s\cdot}} = \{y_{si} : i = 1, \ldots, n\}$, $\boldsymbol{\gamma} = \{\gamma_i : i = 1, \ldots, n\}$, and $\boldsymbol{\omega_{s\cdot}} = \{\omega_{si} : i = 1, \ldots, n\}$ for $s = 1, 2$. With the normal prior $\mathrm{N}(-\tau_s^2/2, \tau_s^2)$, the full conditional for regression coefficients, $\beta_s = (\beta_{s1}, \beta_{s2}, \ldots, \beta_{sP})'$, is derived as,

$$\beta_s | \alpha_s, \boldsymbol{\gamma}, \boldsymbol{\omega_{s\cdot}}, \boldsymbol{\tau^2}, \boldsymbol{y_{s\cdot}} \overset{ind.}{\sim} \mathrm{N}(\xi_s, U_s),$$

where $\xi_s = U_s \Big\{ X' \Big[\Big(\omega_{s1}(\log(\alpha_s^{-1}) - \log(N_{s1}) - \gamma_1) + (y_{s1} - \alpha_s^{-1})/2 \Big), \ldots, \Big(\omega_{sn}(\log(\alpha_s^{-1}) - \log(N_{sn}) - \gamma_n) + (y_{sn} - \alpha_s^{-1})/2 \Big) \Big]' + (-1/2, \ldots, -1/2)' \Big\}$ and $U_s = (X'\Omega_s X + T^{-1})^{-1}$. X is a design matrix consisting of the intercept and the covariates for all samples, that is, $X = (X_1, \ldots, X_n)'$ with $X_i = (1, x_{i2}, \ldots, x_{ip})'$. Ω_s indicates a diagonal matrix of ω_{si}, such that $\Omega_s = \mathrm{diag}(\omega_{s1}, \ldots, \omega_{sn})$. T is also a $P \times P$ diagonal matrix of $\mathrm{diag}(\tau_s^2, \ldots, \tau_s^2)$.

Similarly, the full conditional for the latent factors $\boldsymbol{\gamma}$, with the normal distribution assumption of $\mathrm{N}(-\phi_i^2/2, \phi_i^2)$ with $\phi_i^2 = \phi^2$, is given by

$$(\gamma_1, \ldots, \gamma_n)' | \alpha_1, \alpha_2, \beta_1, \beta_2, \phi^2, \boldsymbol{\omega_{1\cdot}}, \boldsymbol{\omega_{2\cdot}}, \boldsymbol{y_{1\cdot}}, \boldsymbol{y_{2\cdot}} \overset{ind.}{\sim} \mathrm{N}(\zeta, V),$$

where $V = (\Omega_1 + \Omega_2 + \Phi^{-1})^{-1}$ and $\zeta = V \Big\{ \Big[\Big(\big(\omega_{11}(\log(\alpha_1^{-1}) - \log(N_{11}) - X_1'\beta_1) + (y_{11} - \alpha_1^{-1})/2 \big), \ldots, \big(\omega_{1n}(\log(\alpha_1^{-1}) - \log(N_{1n}) - X_n'\beta_1) + (y_{1n} - \alpha_1^{-1})/2 \big) \Big)' + \Big(\big(\omega_{21}(\log(\alpha_2^{-1}) - \log(N_{21}) - X_1'\beta_2) + (y_{21} - \alpha_2^{-1})/2 \big), \ldots, \big(\omega_{2n}(\log(\alpha_2^{-1}) - \log(N_{2n}) - X_n'\beta_2) + (y_{2n} - \alpha_2^{-1})/2 \big) \Big)' \Big] + \Phi^{-1}(-\phi_1^2/2, \ldots, -\phi_n^2/2)' \Big\}$. Φ is a diagonal matrix of $\mathrm{diag}(\phi_1^2, \ldots, \phi_n^2)$.

The full conditional for the auxiliary variables of ω_{si}, with the Pólya-Gamma distribution $\mathrm{PG}(\alpha_s^{-1} + y_{si}, 0)$ prior, takes the form of

$$p(\omega_{si} | \alpha_s, \beta_s, \gamma_i, y_{si}) \propto \exp\Big(-\omega_{si} \big(\log(N_{si}) + X_i'\beta_s + \gamma_i + \log(\alpha_s) \big)^2 / 2 \Big) \mathrm{PG}(\omega_{si} | \alpha_s^{-1} + y_{si}, 0).$$

According to Theorem 1 of Polson *et al.* (2013), this is proportional to a density function of a Pólya-Gamma distribution $\mathrm{PG}\Big(\alpha_s^{-1} + y_{si}, \big(\log(N_{si}) + X_i'\beta_s + \gamma_i + \log(\alpha_s) \big) \Big)$; using the prior conjugacy, the posterior samples of ω_{si} can be taken from the updated Pólya-Gamma distribution.

Other parameters, $(\boldsymbol{\alpha}, \phi^2, \boldsymbol{\tau^2})$, have no closed-form full conditionals with the joint full conditional density

$$\begin{aligned} p(\boldsymbol{\alpha}, \phi^2, \boldsymbol{\tau^2} | \boldsymbol{y}, \beta_s, \boldsymbol{\gamma}) \propto & \Big[\prod_{s=1}^{2} \prod_{i=1}^{n} \frac{\Gamma(y_{si} + \alpha_s^{-1})}{\Gamma(y_{si} + 1)\Gamma(\alpha_s^{-1})} \Big(\frac{\alpha_s^{-1}}{\alpha_s^{-1} + \mu_{si}} \Big)^{\alpha_s^{-1}} \Big(\frac{\mu_{si}}{\alpha_s^{-1} + \mu_{si}} \Big)^{y_{si}} \Big] \\ & \times \mathrm{Exp}(\alpha_1 | a_\alpha) \mathrm{Exp}(\alpha_2 | a_\alpha) \\ & \times \Big[\prod_{i=1}^{n} \mathrm{N}(\gamma_i | -\phi^2/2, \phi^2) \Big] \mathrm{Exp}(\phi^2 | a_{\phi^2}) \\ & \times \prod_{s=1}^{2} \Big[\prod_{p=1}^{P} \mathrm{N}(\beta_{sp} | -\tau_s^2/2, \tau_s^2) \Big] \mathrm{Exp}(\tau_s^2 | a_{\tau^2}). \end{aligned}$$

Hence, the parameters are updated with Metropolis-Hastings steps in MCMC, using log-normal proposal distributions.

For the extended models, the dispersion parameters ϕ_l^2, $l = 1, \ldots, L$, of latent factors are updated using the Metropolis-Hastings algorithm, too. Unlike JNBM_SG, JNBM_Mix has additional parameters $\{\nu_l\}$ and $\{\xi_i\}$. As $\text{Dir}(p_{\nu_1}, \ldots, p_{\nu_L})$ is a conjugate prior for $(\nu_1, \ldots, \nu_L)$, the mixture weight parameters are updated using the Dirichlet distribution with parameters $\big(p_{\nu_1} + |\{i : \xi_i = 1,\ \ i = 1, \ldots, n\}|, \ldots, p_{\nu_L} + |\{i : \xi_i = L,\ \ i = 1, \ldots, n\}|\big)$, where $|A|$ indicates the cardinality of set A. Finally, posterior samples of auxiliary variables $\{\xi_i\}$ can be drawn from the updated discrete probability function: $\Pr(\xi_i = l) \propto \text{N}(\gamma_i| - \phi_l^2/2, \phi_l^2)\nu_l$, $i = 1, \ldots, n$ and $l = 1, \ldots, L$.

As our modeling strategy is taxon-by-taxon, posterior estimates of model parameters for the microbial community (of multiple taxa) can be obtained by repeating the above Gibbs samplers multiple times, but can be done in parallel.

References

Polson, N. G. *et al.* (2013). Bayesian inference for logistic models using pólya–gamma latent variables. *Journal of the American statistical Association*, **108**(504), 1339–1349.
